# CellPyAbility: automated image analysis for high-throughput dose-response screening

**DOI:** 10.1093/bioinformatics/btag513

**Published:** 2026-07-11

**Authors:** James L Elia, Sam Friedman, Ranjit S Bindra

**Affiliations:** Department of Pathology, Yale School of Medicine, New Haven, CT 06510, United States; Yale Center for Research Computing, Yale University, New Haven, CT 06511, United States; Department of Pathology, Yale School of Medicine, New Haven, CT 06510, United States

## Abstract

**Summary:**

Nuclei counting provides a low-cost, metabolic-independent alternative to ATP- or tetrazolium-based cell viability assays. However, the fragmentation of image processing, normalization, and statistical modeling across multiple software platforms hinders high-throughput adoption. We present CellPyAbility, a Python-based suite that automates image processing, dose-response fitting, and synergy analysis. It converts unedited whole-well images into publication-ready graphics in under 1 minute per 96-well plate on standard desktop hardware.

**Availability and implementation:**

CellPyAbility is open-source (MIT License) and available as a Python package via PyPI and Bioconda, or as a code-free application for macOS and Windows. Source code and documentation are available at https://github.com/bindralab/cellpyability and on Zenodo at https://doi.org/10.5281/zenodo.20693745.

## 1 Introduction

Measuring cell viability is fundamental to drug discovery, yet common redox-based assays (e.g. MTT) can be confounded by antioxidants, reactive oxygen species, or transition metals (Maioli *et al.* 2009, Carreño *et al.* 2021, Ghasemi *et al.* 2021). While ATP-based assays (e.g. CellTiter-Glo) are more robust, they are expensive and susceptible to variability between study centers and cell lines (Niepel *et al.* 2019).

Nuclei counting offers a robust alternative with single-cell resolution, compatibility with redox-altering chemicals, and applicability to live cells using non-toxic nuclear dyes. Our research group utilized this method in the discovery of a novel mechanism to overcome drug resistance in glioblastoma (Lin *et al.* 2022, Huseman *et al.* 2024) and the identification of potent synergy between ATR inhibitors and the DNA alkylator temozolomide (Jackson *et al.* 2019).

Further, the method is significantly more affordable; assessing a 96-well plate costs ∼$0.65 USD compared to $16–$30 USD for commercial kits (Script 1, available as supplementary data at *Bioinformatics* online), the difference being multiplicative with the number of replicates, cell lines, and drugs. However, nuclei counting has the high temporal and computational cost of image analysis.

Standard analysis requires a fragmented workflow: image segmentation (e.g. ImageJ, CellProfiler); matrix transformations and normalization in Excel; curve fitting and visualization in proprietary software (e.g. GraphPad Prism). To address this, we developed CellPyAbility, an open-source software suite that automates image processing, dose-response fitting, and synergy analysis for univariate and drug combination cell viability assays, delivering publication-ready graphics from raw microscopy images on commodity hardware.

## 2 Implementation

CellPyAbility is implemented in Python 3 and leverages CellProfiler (Carpenter *et al.* 2006) as a backend for robust nucleus segmentation and modularity. The software is designed for accessibility across different levels of computational expertise, available as both a command-line interface (CLI) for automated workflows and a code-free application for macOS and Windows. CellPyAbility is available on PyPI and Bioconda.

### 2.1 Architecture and modules

**Figure 1:**
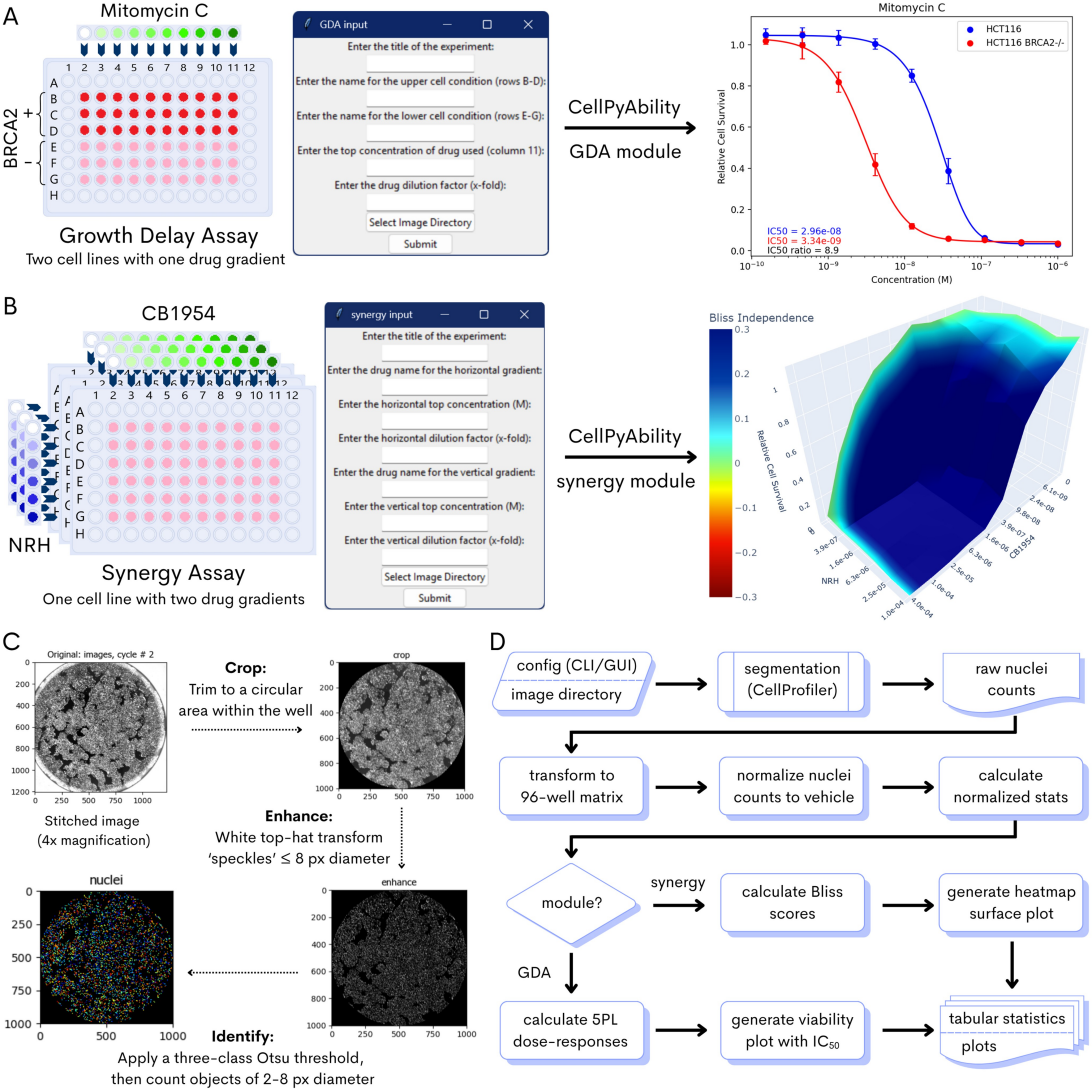
Graphical summaries of CellPyAbility. (A) Left: experimental layout for the chemogenomic use case. Center: code-free application GUI. Right: dose-response plot with IC_50_ calculations. (B) Left: experimental layout for the bioactivation use case. Center: code-free application GUI. Right: 3D surface plot with cell viability (*z*), drug concentrations (*x*, *y*), and Bliss score (heatmap). (C) Image processing and nuclei segmentation pipeline using a 4× microscopy image as an example. (D) Logic flowchart of the CellPyAbility automated analysis for GDA and synergy modules.

The software consists of three primary analysis modules written in Python:

- **Growth Delay Assay (GDA):** This module processes 60 whole-well images (two cell lines in triplicate) to generate univariate dose-response curves (Fig. 1A). It normalizes nuclei counts to vehicle controls, fits data using 5-parameter logistic (5PL) regression (Gottschalk and Dunn 2005), which accounts for asymmetry in the transition region better than 4PL models, and determines the IC_50_ via inverse prediction of the fitted 5PL model at 50% vehicle-normalized cell survival.
- **Synergy:** This module analyzes 180 images to assess drug combinations (Fig. 1B). It utilizes simultaneous drug gradients to compute a 3D surface map of cell viability and Bliss independence scores, a standard metric for drug synergy defined as $\mathrm{Bliss}={SF}_{A}\times {SF}_{B}-{SF}_{AB}$ where ${SF}_{A}$ and ${SF}_{B}$ are the surviving fractions of the individual drugs, and ${SF}_{AB}$ is the observed surviving fraction of the combination (Bliss 1939). The score is calculated for each combination (59 total).
- **Simple:** This module produces a raw nuclei-count matrix for users requiring maximum flexibility for downstream analysis.

The Python dependencies are minimal: matplotlib, numpy, pandas, plotly, and scipy.

### 2.2 Image processing pipeline

CellPyAbility utilizes a CellProfiler pipeline to process stitched microscopy images. The default pipeline performs three key transformations: cropping the image to the well interior, applying a white top-hat transform to enhance nuclei and reduce background, and applying a three-class Otsu threshold to segment and count nuclei (Fig. 1C), where the middle class is considered foreground. This approach is robust to variability in cellular morphology and technical noise across diverse cell lines, achieving a precision of 0.984 and recall of 0.924 across 1464 nuclei from 15 randomly cropped images of HCT116, LN229, or U2OS, which possess diverse morphologies (Table S1, available as supplementary data at *Bioinformatics* online).

While using CellProfiler adds an external dependency, the familiar image analysis ecosystem and easy modularity are key advantages. For example, if images are 10× magnification (instead of the default 4×), the pixel width of the average nucleus can be directly adjusted in a code-free user interface.

### 2.3 Batch implementation

Using a provided configuration file, users can automate the image processing and analysis of multiple experiments. This batch processing capability allows for the analysis and visualization of >50 GDAs in under 1 hour and seamlessly handles GDA and synergy experiments within a single batch.

## 3 Results

The utility of CellPyAbility was assessed on computational efficiency and biological accuracy. Following a performance evaluation on standard hardware, we demonstrate the software’s application in real-world scenarios, validating the GDA and Synergy modules through established DNA damage and bioactivation screens.

### 3.1 Performance and features

CellPyAbility significantly accelerates analysis time. The GDA module processes 60 unedited images and generates fitted curves in under 1 minute on commodity hardware (Intel Core i7 @ 2.8 GHz, 16 GB RAM). The Synergy module processes 180 images and computes 3D synergy maps in under 3 minutes. When applicable, we leverage vectorized NumPy operations to minimize computational overhead. In addition to publication-ready graphical outputs, the software provides tabular data at every step (raw counts, normalized matrices, and statistics) to facilitate custom analysis and visualization. This represents a significant efficiency gain over manual workflows, which often require data wrangling across three distinct software packages (image analysis, spreadsheet, and graphing software) while generating effectively identical results (Fig. S1A-D, available as supplementary data at *Bioinformatics* online).

### 3.2 Use case: chemogenomic interactions in cancer

We validated the GDA module by replicating a known chemogenomic interaction between *BRCA2* deficiency and sensitivity to the DNA interstrand crosslinking agent mitomycin C (MMC) (Xia *et al.* 2006). Using CellPyAbility, we observed a nine-fold decrease in IC_50_ in *BRCA2*-deficient HCT116 cells compared to wild-type, accurately recapitulating the expected phenotype (Fig. S1A, available as supplementary data at *Bioinformatics* online). Unlike proprietary tools such as GraphPad Prism, which require manual data wrangling across multiple software packages, CellPyAbility generated these metrics directly from the image directory.

### 3.3 Use case: bioactivation synergy

We demonstrated the utility of the Synergy module by analyzing the interaction between the prodrug CB1954 and its bioactivating cofactor, nicotinamide riboside hydride (NRH) (Knox *et al.* 1991). The software successfully identified a potent synergistic interaction, visualizing a 1000-fold shift in IC_50_ via an interactive 3D surface plot (Fig. S1C available as supplementary data at *Bioinformatics* online). This streamlines the workflow compared to existing standalone tools like Combenefit (Di Veroli *et al.* 2016), which require pre-quantified tabular data inputs rather than raw images.

## 4 Discussion

CellPyAbility democratizes access to high-throughput screening by removing the barrier of complex data analysis. By combining the low cost of nuclei counting with the speed of automated processing, the software enables academic labs to perform screens previously restricted to core facilities.

Current limitations include the requirement for adherent cells, as the image analysis relies on a single focal plane. While suspension cells can be adhered via centrifugation or coating, this requires protocol adaptations not yet validated in the software. While the use of specific formats is intended to increase ease of use, it also results in rigid input requirements; e.g. 60 images for one plate, which is a translation of the 60 inner wells of a 96-well plate, a standard practice to avoid edge effects. Future versions of CellPyAbility could increase the flexibility of accepted inputs while still maintaining high automation by accepting a “plate map” feature as a command-line file path or through an interactive GUI.

In conclusion, CellPyAbility provides a fast, open-source, and robust alternative to commercial viability analysis suites. By standardizing the workflow from raw images to statistical analysis, it improves reproducibility and facilitates the screening of novel therapeutic combinations.

## Supplementary Material

btag513_Supplementary_Data

## Data Availability

The data and source code underlying this article are available on GitHub (https://github.com/bindralab/cellpyability) and Zenodo (https://doi.org/10.5281/zenodo.20693745).
