# Supplementary material for: CellPyAbility: automated image analysis for high-throughput dose-response screening
Source: Bioinformatics. 2026 Jul 11;42(7):btag513. doi: 10.1093/bioinformatics/btag513 (PMC13401477; doi:10.1093/bioinformatics/btag513)
Supplement: btag513_Supplementary_Data [file btag513_supplementary_data.zip › CellPyAbility_SI.docx]

CellPyAbility: automated image analysis for high-throughput dose-response screens

Supplementary Information


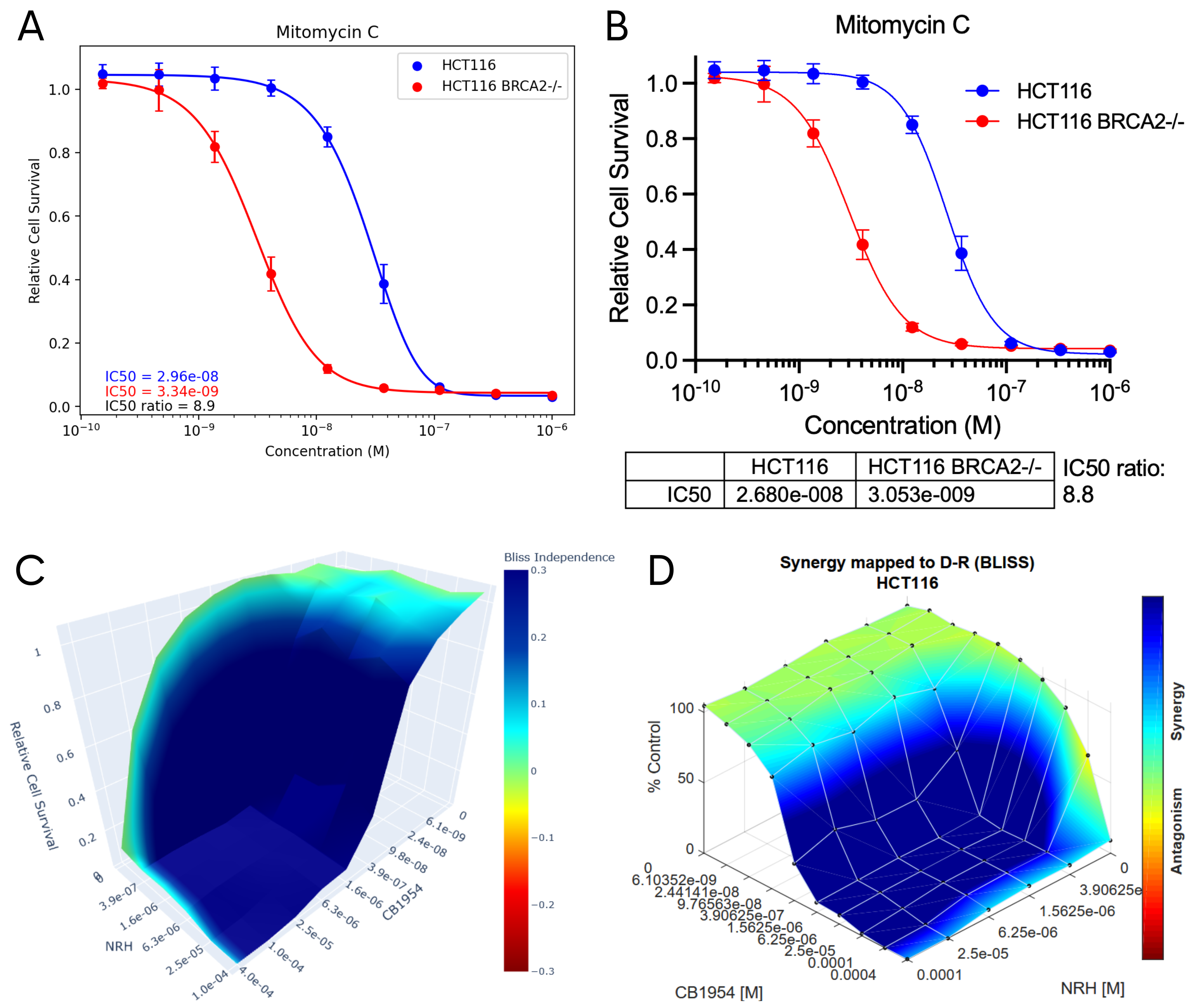


**Supplemental Figure 1: CellPyAbility analysis method comparison. A**) Fully automated CellPyAbility GDA output for HCT116 ± BRCA2 treated with mitomycin C. **B**) Manual Excel and GraphPad Prism output for HCT116 ± BRCA2 treated with mitomycin C. **C**) Fully automated CellPyAbility synergy output for HCT116 with CB1954 and NRH. **D**) Partially automated Combenefit Bliss synergy output for HCT116 with CB1954 and NRH.

| **cell line** | **drug** | **TP** | **FP** | **FN** | **precision** | **recall** |
| --- | --- | --- | --- | --- | --- | --- |
| HCT116 | mitomycin C | 148 | 3 | 12 | 0.9801 | 0.925 |
| HCT116 | mitomycin C | 162 | 4 | 16 | 0.9759 | 0.9101 |
| HCT116 BRCA2-/- | mitomycin C | 53 | 3 | 7 | 0.9464 | 0.8833 |
| HCT116 BRCA2-/- | mitomycin C | 6 | 0 | 0 | 1 | 1 |
| HCT116 | mitomycin C | 26 | 2 | 5 | 0.9286 | 0.8387 |
| LN229 Cas9 | JH292 | 146 | 1 | 11 | 0.9932 | 0.9299 |
| LN229 Cas9 | JH292 | 136 | 2 | 10 | 0.9855 | 0.9315 |
| LN229 Cas9 | JH843 | 153 | 2 | 9 | 0.9871 | 0.9444 |
| LN229 Cas9 | JH843 | 113 | 0 | 5 | 1 | 0.9576 |
| LN229 Cas9 | JH292 | 58 | 1 | 13 | 0.9831 | 0.8169 |
| U2OS TRIPZ shBRCA2 | talazoparib | 179 | 3 | 9 | 0.9835 | 0.9521 |
| U2OS TRIPZ shBRCA2 + dox | talazoparib | 94 | 1 | 6 | 0.9895 | 0.94 |
| U2OS TRIPZ shBRCA2 + dox | talazoparib | 32 | 0 | 6 | 1 | 0.8421 |
| U2OS TRIPZ shBRCA2 | talazoparib | 45 | 0 | 0 | 1 | 1 |
| U2OS TRIPZ shBRCA2 | talazoparib | 2 | 0 | 2 | 1 | 0.5 |
| **total** |  | 1353 | 22 | 111 | 0.984 | 0.924 |

**Supplemental Table 1: CellPyAbility default image analysis precision and recall.** 100x100 px crops were randomly selected from diverse data including three backgrounds of unique morphology, two isogenic pairs, four drug treatments, and five cell densities per background. TP = true positive (is nucleus, segmented), FP = false positive (is not nucleus, segmented), FN = false negative (is nucleus, not segmented). Ground truth annotations by a blinded human expert.
