# Supplementary figures and images for: CellPyAbility: automated image analysis for high-throughput dose-response screening
Source: Bioinformatics. 2026 Jul 11;42(7):btag513. doi: 10.1093/bioinformatics/btag513 (PMC13401477; doi:10.1093/bioinformatics/btag513)

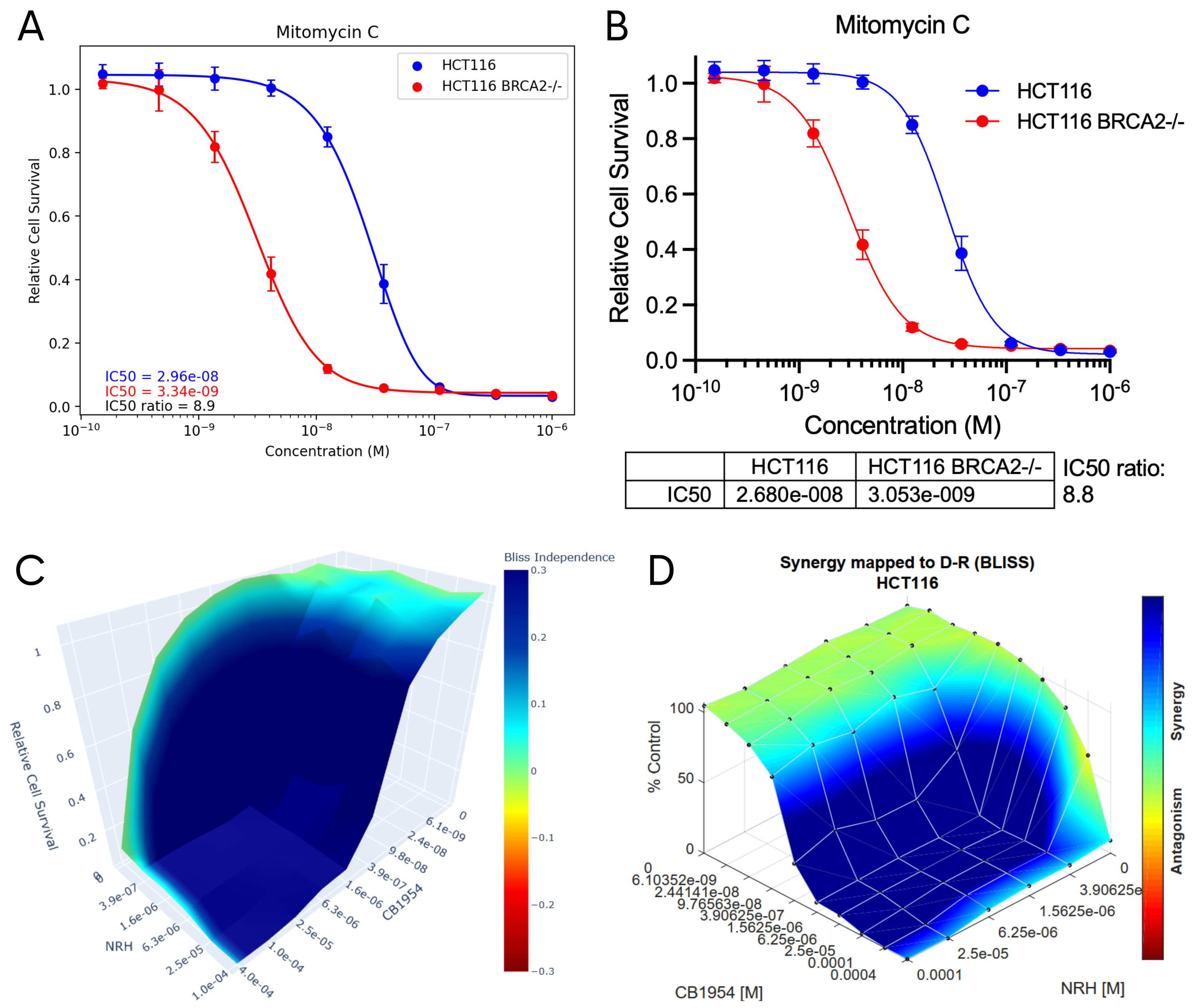

Supplement: btag513_Supplementary_Data [file btag513_supplementary_data.zip › CellPyAbility_Bioinformatics_SuppFig1.png]
